# The Impact of Non-normative Displays of Emotion in the Workplace: How Inappropriateness Shapes the Interpersonal Outcomes of Emotional Displays

**DOI:** 10.3389/fpsyg.2020.00006

**Published:** 2020-02-14

**Authors:** Arik Cheshin

**Affiliations:** Department of Human Services, University of Haifa, Haifa, Israel

**Keywords:** emotion display, interpersonal effects of emotion, social influence of emotion, inappropriateness, incivility

## Abstract

When it comes to evaluating emotions as either “good” or “bad,” everyday beliefs regarding emotions rely mostly on their hedonic features—does the emotion *feel* good to the person experiencing the emotion? However, emotions are not only felt inwardly; they are also displayed outwardly, and others’ responses to an emotional display can produce asymmetric outcomes (i.e., even emotions that feel good to the displayer can lead to negative outcomes for the displayer and others). Focusing on organizational settings, this manuscript reviews the literature on the outcomes of emotional expressions and argues that the evidence points to perceived (in)appropriateness of emotional displays as key to their consequences: emotional displays that are deemed inappropriate generate disadvantageous outcomes for the displayer, and at times also the organization. Drawing on relevant theoretical models [Emotions as Social Information (EASI) theory, the Dual Threshold Model of Anger, and Asymmetrical Outcomes of Emotions], the paper highlights three broad and interrelated reasons why emotion displays could be deemed unfitting and inappropriate: (1) characteristics of the displayer (e.g., status, gender); (2) characteristics of the display (e.g., intensity, mode); and (3) characteristics of the context (e.g., national or organizational culture, topic of interaction). The review focuses on three different emotions—anger, sadness, and happiness—which differ in their valence based on how they feel to the displayer, but can yield different interpersonal outcomes. In conclusion, the paper argues that inappropriateness must be judged separately from whether an emotional display is civil (i.e., polite and courteous) or uncivil (i.e., rude, discourteous, and offensive). Testable propositions are presented, as well as suggested future research directions.

People tend to think of emotions as either positive or negative. Likewise, valence—whether an emotion is experienced as positive or negative—is a fundamental basis for classifying emotions in the literature on emotion (e.g., Russell, 1980). Much of this literature is concerned with the antecedents and consequences of valence for the person *experiencing* an emotion—i.e., whether a given emotion is experienced as pleasant or unpleasant. When *expressed* emotions are examined in the context of interpersonal interactions, an interesting and complex dynamic comes into play, in which the effects of an emotional display are shaped not only by the emotion’s valence but also by its (in)appropriateness for the situation. Inappropriateness entails a mismatch between what is perceived as normative in a particular context and what is actually displayed (Ekman, 1993; Shields, 2005; Geddes and Callister, 2007). Thus, even a positively valenced emotion such as happiness could have adverse outcomes for the displayer and other parties (including the organization) if the happiness is perceived as being displayed in an inappropriate manner (e.g., when a service provider smiles happily in response to a customer’s complaint about poor service).

Taking an organizational perspective, the present study reviews recent findings on the interpersonal dynamics of emotion in the workplace (e.g., van Kleef et al., 2016) and, in particular, findings on how inappropriateness in an emotional display affects outcomes for both the displayer and the organization. This work builds on and extends recent research into the asymmetrical effects of emotion, when so-called positive emotions lead to negative outcomes and vice versa (Lindebaum and Jordan, 2011, 2014; van Kleef, 2014). In addition, the review draws on two other theoretical frameworks: Emotions as Social Information (EASI) theory (van Kleef, 2010, 2016; van Kleef et al., 2012) and the Dual Threshold Model of Anger (Geddes and Callister, 2007).

Perceptions of (in)appropriateness are informed by prevailing norms and expectations concerning emotional expressions, which are referred to as display rules (e.g., Ekman, 1993; Shields, 2005). Display rules dictate emotion display expectations for a particular role or status and/or a given context (Matsumoto, 1990; Shields, 2005; Diefendorff et al., 2010; Moran et al., 2013). Such rules determine what is considered appropriate in terms of the valence of the emotion(s) displayed (positive or negative) as well as other aspects of the display (e.g., its intensity and duration), usually with reference to specific discrete emotions. Various elements of the display can combine to shape perceived inappropriateness, including characteristics of the displayer, such as status or gender; characteristics of the display, such as its intensity or display mode (e.g., face-to-face vs. computer-mediated communication); and characteristics of the context, whether broad (e.g., culture) or specific (e.g., the nature of the task or issue at hand).

To allow for an in-depth look at the interplay between valence and inappropriateness on the effects of emotional displays in the workplace, this paper examines work on three emotions—anger, sadness, and happiness. These emotions are interesting because while they are basic, “core” emotions with defined valences (negative for anger and sadness, positive for happiness; e.g., Russell, 1980), their effects may differ depending on whether one takes an intrapersonal or interpersonal perspective. For instance, while *experiencing* sadness is unpleasant (negative valence), *displaying* sadness may lead to the positive experience of receiving comfort from others (a positive outcome) (Hendriks et al., 2008). At the same time, discrete emotions allow for a clean and unclouded examination of whether a given emotional display is inappropriate—i.e., the degree to which it violates accepted norms and rules—and, therefore, the degree to which inappropriateness impacts the response to (i.e., outcome of) an emotional display.^1^ The literature provides mixed findings regarding the outcomes of emotional displays involving anger, sadness, and even happiness. There are times where such displays lead to positive outcomes for the displayer and/or the organization, while at other times they lead to negative outcomes. Displays of anger, for example, have been found to benefit the displayer in negotiation settings (van Kleef et al., 2004), but Lewis (2000) found that leaders displaying anger were assessed as less effective. In accordance with Lewis’s findings, Madera and Smith (2009) found that leaders who displayed sadness in times of crisis were assessed more favorably than those that displayed anger. However, medical students who displayed deep sadness (by crying) were ridiculed and deemed unprofessional (Wagner et al., 1997). As for happiness, smiling service providers have more satisfied customers (Barger and Grandey, 2006); yet, in another study, individuals who appeared (too) happy were assessed as more gullible and were exploited (Barasch et al., 2016). Thus, there seem to be no clear patterns for the outcomes of emotional displays (interpersonal impact) based solely on the valence experienced (intrapersonal impact).

One conclusion that arises even from this very brief survey is that, as noted by Lindebaum and Jordan (2014), there are times when feeling bad is good, and feeling good is bad. They call for greater study of asymmetric relationships between emotions and their consequences. Building upon this argument and that of Lindebaum et al. (2016) that the outcomes of anger expressions are determined in part by the (in)appropriateness of the display, this review extends this line of thought to include other emotions (happiness and sadness), highlighting inappropriateness as a central determinant of the outcome (positive or negative).

More precisely, the present theoretical article is prompted partly by burgeoning interest in how anger displays can have contradictory outcomes based on whether or not they are perceived as appropriate (e.g., Geddes and Callister, 2007; van Kleef and Côté, 2007; Adam et al., 2010; Lindebaum et al., 2016; Stickney and Geddes, 2016; Callister et al., 2017; Glikson et al., 2019). This review also builds on an established body of work showing that happiness is seen as the only emotion appropriate for display by service providers (e.g., Hochschild, 1983; Rafaeli and Sutton, 1987; Grandey et al., 2015; Sayre et al., 2019). Yet, despite its obvious presence in the workplace, sadness has received relatively less attention. One goal of this work is to highlight the commonalities in how perceived (in)appropriateness of an emotional display affects individual and organizational outcomes, whether the emotion at issue is anger, happiness, or sadness.

This paper proceeds as follows. The next few sections provide background and define relevant terms. This is followed by a review and a proposed model. In the course of the review, seven testable propositions are presented. The paper concludes with a claim that inappropriateness has two different forms that dictate the process of the response to the emotional display.

## Inappropriateness

Human beings experience emotions not only directly but also through a meta-emotional lens (Lundh et al., 2002; Shields, 2005). That is, people do not merely experience emotions, but evaluate emotions’ social impact, including assessing whether a given emotion is appropriate to display in a particular situation. To put it differently, people develop norms and expectations for emotional displays, and as such, to be socially competent means adhering to these norms and expectations (Zawadzki et al., 2013). These meta-emotional evaluations of the (in)appropriateness of emotions occur both on an individual, intrapersonal level, and on a social, interpersonal level. For example, someone who expressed anger in an inappropriate manner might later, while assessing the incident, feel guilt or shame for crossing the line (Gottman et al., 1997). The present paper is not concerned with such intrapersonal responses, but only with interpersonal judgments as to whether an emotional display is normative or deviant—including both the antecedents of inappropriateness (i.e., what determines whether another’s emotion display is perceived as inappropriate) and the consequences of this meta-emotional evaluation for the displayer and for the organization.

Jaggar (1989) referred to individuals who display emotions that are inappropriate or atypical as “emotional outlaws.” And indeed, individuals who display emotions in a way that deviates from the norm may be treated like outlaws, for instance being shunned, stigmatized, or marginalized (Thoits, 1985, 2004; Clark, 1987).^2^ Shields (2005) points out that appropriateness in emotional displays is judged by (1) qualitative fit—whether the correct emotion is displayed; (2) quantitative fit—whether the intensity or magnitude of the emotion displayed is both necessary (not too high) and sufficient (not too low); and (3) compatibility with existing standards—whether the display is in tension with expectations about the emotional experiences and expressions suitable for a given person or situation. Shields also notes that all discussions of appropriateness in emotional displays are political, in the sense that it is usually groups with more political power that dictate what is normative. For example, because the business world is male-dominated, emotional expressions in the business world reflect traditionally masculine perceptions and expectations, and women in such contexts may need at times to mask their true feelings so as to match the emotional norm. The model presented in this manuscript is based on these insights and builds on them to further understand the role of inappropriateness in emotional displays and their consequences in the workplace.

One emotion theory that deals directly with issues of appropriateness is the Dual Threshold Model of Anger (Geddes and Callister, 2007). This model suggests two thresholds, or boundaries, which define when expressions of anger are regarded as acceptable: the *expression threshold*, below which anger is suppressed and not displayed to others, and the *impropriety threshold*, above which the display is considered improper. According to the model, only anger displays between the expression threshold and the impropriety threshold are thought to be appropriate and normative. These anger displays serve a purpose (e.g., informing people that an apparent injustice has been committed or that a goal has been frustrated), and they should yield positive outcomes—including, in the best case, a resolution of the problem that caused the anger. In contrast, anger that crosses the impropriety threshold is likely to yield negative outcomes for the displayer, and to leave the problem that caused the anger unaddressed. The current theoretical paper builds on the Dual Threshold Model of Anger and suggests that the logic regarding the impropriety threshold applies not only to anger, but to all emotional displays.

Another theory on which the current model is based is the Emotion as Social Information (EASI) theory (van Kleef, 2010, 2016). EASI suggests two routes by which displayed emotions influence those who observe them—the *affective route* and the *inferential route*. The affective route concerns the impact of displayed emotions on the emotions of others and includes processes such as emotional contagion (e.g., Barsade, 2002; Cheshin et al., 2011) and emotional response (e.g., Hareli and Rafaeli, 2008). The inferential route concerns the impact of displayed emotions on others’ constructed evaluations or appraisals regarding the situation or the displayer (e.g., Hareli and Hess, 2010). Both the affective and the inferential routes impact behaviors or responses to emotional displays among targets or observers of the emotion. EASI postulates that emotional expressions may have disadvantageous consequences for the expresser to the degree that they are perceived as inappropriate for the context and that this process occurs primarily via the affective route. Specifically, the EASI model argues that inappropriate displays elicit mainly negative affective responses (van Kleef et al., 2012; van Kleef, 2014). The present work suggests that *inferential* processes are also impacted by the (in)appropriateness of emotional displays, and that inferences drawn from the emotional display serve alongside emotions elicited by the display to determine the difference between a positive and negative response.

### Display Rules—Delimiting Appropriate Emotion Expression at Work

In the organizational context, emotion display rules refer to expectations regarding appropriate emotional expressions at work, including what emotions should be expressed, how, and when (Rafaeli and Sutton, 1987; Morris and Feldman, 1996; Grandey and Gabriel, 2015). As such, display rules are similar to etiquette—a set of conventions or codes dictating how one should behave in social interactions (Friedman and Miller-Herringer, 1991). Adhering to display rules is considered a specific in-role expectation (Diefendorff et al., 2006). Display rules have been tied mostly to service work (Hochschild, 1983), but specific display rules have been found across a range of professions—from funeral directors (e.g., Ashforth and Humphrey, 1993) to flight attendants (e.g., Hochschild, 1983), bill collectors (e.g., Rafaeli and Sutton, 1991), convenience store clerks (Rafaeli, 1989), and contestants in beauty pageants (e.g., DePaulo, 1992). Worldwide, display rules in service jobs tend to demand that employees show positive emotions—“service with a smile”—and hide negative ones (Wharton and Erickson, 1993; Grandey et al., 2010). However, the prevailing norm in organizations (at least in Western societies) is to keep even positive emotions in check and relatively controlled (Kramer and Hess, 2002). Failure to adhere to such display rules is considered unprofessional.

Display rules are there for a purpose. They have been shown to improve the satisfaction of target customers or audiences, and help in creating a desired emotional climate (Gabriel et al., 2016). For employees, the need to constantly display positive emotions regardless of what one is feeling can be a strain, requiring the employee to invest effort in emotion regulation or what has been termed emotional labor (e.g., Hochschild, 1983; Diefendorff and Gosserand, 2003; Grandey, 2015). However, even when emotions are manipulated and not necessarily authentic, they can yield positive outcomes for the displayer as long as they are deemed to be the appropriate emotions for the situation (e.g., Clark and Taraban, 1991; Cheshin et al., 2018).

## Review and Model Overview

**FIGURE 1:**
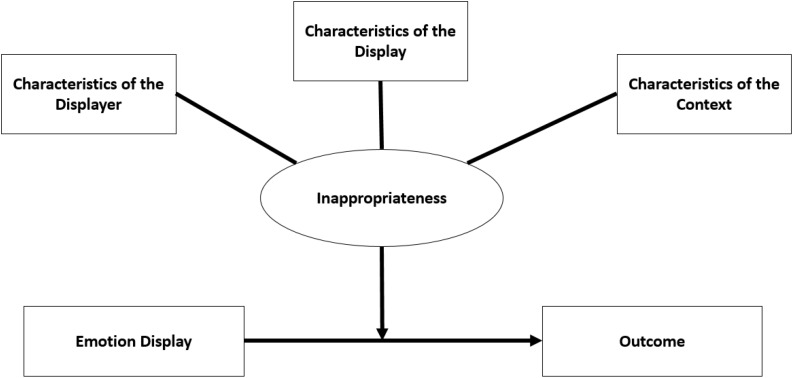
Theoretical model.

This paper focuses on research dealing with displayed emotions in organizational settings. The criteria for inclusion in the review were 2-fold: reviewed papers (1) dealt with the (in)appropriateness of emotional displays at work, and (2) focused on anger, happiness, and/or sadness. The proposed model draws on this literature and, in particular, on three existing theoretical frameworks: The Dual Threshold Model of Anger, the EASI model, and the asymmetrical outcomes of emotion displays. The goal of the model is to describe the characteristics of inappropriateness and how they shape the outcomes of emotional displays at work for (1) the displayer and (2) the organization. The theoretical model is presented in Figure 1.

The sections below provide a general discussion of emotion displays and describe what is meant by outcomes. They are followed by the heart of this paper: three sections dealing with the characteristics of inappropriateness—(1) the display, (2) the displayer, and (3) the context. This discussion leads to seven testable propositions.

### Emotion Displays

Emotions are outwardly displayed in numerous ways, including facial expressions (e.g., Ekman and Friesen, 1976; van der Schalk et al., 2011; Jack et al., 2016), gestures and body language (e.g., Atkinson et al., 2004; Dael et al., 2012; de Gelder et al., 2015), and attributes of the voice (e.g., Banse and Scherer, 1996; Sauter et al., 2010; Cowen and Elfenbein, 2018). Emotions can also be conveyed textually, without the presence of the person experiencing the emotion (e.g., Dresner and Herring, 2010; Cheshin et al., 2011; Gettinger and Koeszegi, 2015). These expressions and displays are recognized cross-culturally (e.g., Elfenbein and Ambady, 2002). Importantly, the social effect of these various modes of emotion displays is considered functionally equivalent, meaning that an emotion display will be perceived as representing the same emotion whether it is displayed via the face, body, tone of voice, textually, or with symbols such as emojis, and will have the same interpersonal effect^3^ (van Kleef et al., 2012; van Kleef, 2017). Yet, the outcomes for these displays will be governed by whether or not they are judged as (in)appropriate.

With respect to the interpersonal nature of emotion displays, the question of authenticity must be mentioned. It is well-established that displays of emotion may not be authentic representations of the displayer’s feelings, but a modulated response or surface act (e.g., Gross, 1998; Grandey and Sayre, 2019). It has also been shown that observers can generally distinguish between real and manipulated or fake emotion displays (Okubo et al., 2012; Côté et al., 2013; Hideg and van Kleef, 2017). These questions, while important, are not of concern in the present paper, where at issue is the response to inappropriate displays of emotion and not the authenticity of the display. That is, this review assumes that even a fake, inauthentic emotion display, regardless of its valence, will also be judged as to whether or not it is inappropriate.

### Outcomes of Inappropriate Emotional Displays

The interpersonal outcomes of emotional displays are far-reaching. They can include outcomes not only for the displayer but also for the relationship between the displayer and the target, or for the organization. The end result of the EASI model is a response to an emotional display of another person. For example, one possible outcome could be forfeiting or giving in to an opponent in a negotiation (van Kleef et al., 2004). In the present work, a broad view is taken, considering not only outcomes that manifest as behavior but also outcomes that remain at the level of attitudes toward or assessments of the displayer (inferences in the terminology of EASI), or emotional effects in the target or observer (affective responses in EASI’s terms), as well as the implications for the organization. Here, I take my cues from the Dual Threshold Model of Anger (Geddes and Callister, 2007), which is concerned with how an emotional display helps or hinders the goals of a team or organization. Since the issue here is inappropriate displays of emotion, outcomes for both the individual and the organization are mostly negative. This leads to the main argument of this article:

Proposition 1: Displays of emotion, when deemed inappropriate, will lead to a negative outcome for the displayer and the organization.

## Characteristics of Inappropriateness

Three broad elements of an emotional display can combine to shape perceived inappropriateness. These are (1) characteristics of the displayer, such as status or gender; (2) characteristics of the display, such as its intensity or mode; and (3) characteristics of the context, whether narrow, such as the topic of the interaction, or broader, such as organizational or national culture. Clearly, these are interrelated and overlapping (e.g., characteristics of the displayer may interact with characteristics of the display to determine its inappropriateness in a given context), yet for simplicity and clarity, I will discuss each one separately. The three sections that follow do not claim to delineate each element clearly and cleanly, but rather use each one in turn as a lens through which to examine the question at hand.

## Characteristics of the Displayer

Displayer characteristics can lead to differing expectations regarding emotion displays. For example, people may have different sets of expectations for emotional expressions by a boss or political leader compared with a worker of lower status. These expectations shape evaluations as to when an emotional display is or is not appropriate for a person in a specific role or status. Much of the literature on how characteristics of the displayer affect the perceived inappropriateness of emotional displays focuses on two such attributes: status and gender. This section summarizes the main findings of that literature.

### Status

From an organizational perspective, status is a person’s position in the organizational hierarchy. With higher status come “emotional privileges” (Averill, 1982; Geddes et al., 2019), meaning that individuals with higher status in the organization are afforded more freedom in some aspects of emotional expression. That is, emotion displays that might be deemed inappropriate for a subordinate may be regarded as appropriate for a superior. For example, it has been shown that despite feeling and experiencing more anger in the workplace, lower-status workers are less likely to express anger (Sloan, 2004). Likewise, Callister et al. (2017) found that supervisors have more “space” between the expression threshold and the impropriety threshold “to express anger without being labeled as, or sanctioned for, deviant, inappropriate anger expression. Subordinates, on the other hand, with lower status, do not share this same emotional privilege, have less space between thresholds, and are more likely to be sanctioned when expressing anger, especially to their boss” (p. 70). For their part, individuals in high-status positions are likely to justify their own displays of anger on the grounds that these are good for the organization as a whole and therefore are not only appropriate, but are also warranted (Fitness, 2000; Callister et al., 2017).

Because higher-status individuals are granted greater leeway to express anger, anger expressions also provide cues regarding status. Tiedens (2001) showed that when other information was not available, job candidates, co-workers, and politicians were deemed of higher status when they displayed anger. Other scholars have also found that expressions of anger increase perceptions of power and control, which are signifiers of status (Conway et al., 1999; Domagalski and Steelman, 2007). Moreover, managers in construction work who displayed anger were seen as more effective leaders (Lindebaum and Fielden, 2011).

Interestingly, however, even those who are higher in status are not universally immune from the impact of inappropriateness. For example, even high-status figures such as the president of the United States are impacted by the (in)appropriateness of emotional displays, as was demonstrated by Bucy (2000). Participants who were presented with non-verbal emotional responses of then-President Bill Clinton to news events were asked to evaluate the president. These emotional responses were manipulated to be either appropriate or not for the news event depicted. Emotional displays that were inappropriate to the situations led observers to feel negative emotions toward the president and also led them to make negative trait evaluations of him.

At times, the outcome of an emotional display is not clear-cut, with different individuals holding different interpretations of the episode’s effects or meaning. Status (among other things) may affect attitudes toward the consequences of an anger display as well as toward its (in)appropriateness. For instance, in some studies, supervisors who expressed anger seemed to believe that their anger expressions led to a positive outcome, however, unbeknownst to the supervisors, subordinates’ respect for the supervisor and trust in the relationship suffered (Fitness, 2000; Callister et al., 2017). Thus, even when the outcome of an emotion display is perceived as beneficial by the displayer, there may be hidden costs that could have effects down the line. However, there is also evidence that this dynamic is affected by the degree to which the anger is deemed inappropriate. Koning and van Kleef (2015) found that inappropriate anger led to less trust in and respect for the leader who displayed the anger, and also led to less organizational citizenship behavior (i.e., subordinates’ willingness to engage in extra work beyond their assigned roles). In contrast, when the anger was deemed appropriate, subordinates’ organizational citizenship behavior did not suffer.

Although far less work has been conducted on status and displays of sadness and happiness than for anger, there is some research showing that expectations and norms for expressions of sadness and happiness differ based on status. For example, expressions of sadness are believed to be more normative for low-status individuals compared to those of high status, while happiness expressions are believed to be less appropriate for low-status individuals compared to those of higher status (Conway et al., 1999).

To establish this point more compellingly, future work could explicitly test the effects of the same emotional display by people of different status (and not merely the interpretation of these displays as more or less inappropriate). Such studies could examine whether or not emotional displays indeed lead to different outcomes based on the status of the displayer. Based on current evidence, I propose the following:

Proposition 2: The displayer’s status will impact perceptions of an emotional display as inappropriate, such that emotional displays by people of lower status will be perceived as inappropriate compared to displays of people of higher status.

### Gender

When it comes to gender, many social contexts involve clear expectations about emotional displays. Broadly speaking, Western societal norms imply that even the same emotional displays are assessed differently depending on whether the person expressing the emotion is male or female. For example, in their paper succinctly titled “She’s Emotional. He’s Having a Bad Day,” Barrett and Bliss-Moreau (2009) showed that men’s displays of emotion are given situational attributions, whereas those of women are given dispositional attributions. Brescoll and Uhlmann (2008) found a similar pattern with respect specifically to anger. Thus, emotional displays may be interpreted differently for men and women from the outset.^4^

The phenomenon of prescribed societal gender roles regarding emotions—even for very young children (Brody, 2000)—is well documented and needs only brief mention here. Women are expected to be more caring and tender and to express their emotions more openly than men (e.g., Shields, 2005). Moreover, it has been found that women’s motives for emotional regulation are relationship oriented, while men’s are power based (Timmers et al., 1998). That study further found that individuals deliberately regulate their emotional displays so as “to avoid gender-inappropriate emotional impressions” (p. 975).

With respect specifically to the emotions examined in this paper, happiness and sadness are considered normative for females more than males, while anger is considered normative for males more than females (Timmers et al., 1998; Ragins and Winkel, 2011; Sloan, 2012). If women do express anger, they are expected to do so indirectly and passively, while men’s anger displays are expected to be direct and even aggressive. Domagalski and Steelman (2007) claim that these differences are not so stark in the work setting. Nonetheless, it is a consistent finding that female leaders who display anger in organizational settings are penalized (e.g., they receive worse evaluations from their colleagues), while male leaders who display anger are not (Lewis, 2000; Ragins and Winkel, 2011). Brescoll and Uhlmann (2008) found that expressions of anger reduced attributions of status to women regardless of their actual organizational status or rank. Gibson et al. (2009) also found that female displays of anger in organizations are less likely to result in positive outcomes compared to those of males.

Salerno and Peter-Hagene (2015) used a juror decision task to examine the effect of anger displays by males vs. females. They presented participants with a scenario where one member of a jury angrily opposed an otherwise unanimous decision. Participants were more likely to reconsider their decision and change their vote if the angry individual was male rather than female. These findings add to the evidence that women are more likely to be labeled “emotional outlaws” and assessed as displaying emotions that are inappropriate (Shields, 2005). Thus, I propose the following:

Proposition 3: The Displayer’s gender will impact perception of an emotional display as inappropriate, such that displays of anger by females will be perceived as more inappropriate than such displays by males, whereas displays of sadness and happiness by males will be perceived as more inappropriate than such displays by females.

Gender affects evaluations of emotional displays not only in relation to the specific emotion expressed but also in relation to the characteristics of the display. For example, females’ emotional displays at work are more likely to be assessed as being of an inappropriate level of intensity (Ragins and Winkel, 2011). Moreover, gender differences have been found in the use of emotions and emojis (Wolf, 2000; Chen et al., 2017), where women were found to be more likely to use those digital displays of emotion. This leads to the next section.

## Characteristics of the Display

In addition to differences in attributes of the displayer, when it comes to (in)appropriateness, differences can also arise in the manner by which emotions are conveyed. These differences may involve technical or formal features, such as the display medium (e.g., whether emotions are displayed via the phone or a computer), or attributes of the display itself (e.g., intensity). We will begin with the latter.

### Intensity

Emotions are experienced and expressed at various strengths and magnitudes (Frijda et al., 1992; Sonnemans and Frijda, 1994). Differences in the intensity of felt emotions reflect the importance of the trigger (i.e., the event giving rise to the emotion) for the individual (Ortony et al., 1988; Clore, 1994; Heylen et al., 2015). For instance, people become angrier when an important goal is frustrated compared to a less essential goal. In the normal course of things, such differences in intensity are also apparent in expressed emotions (e.g., Banse and Scherer, 1996; Cheshin et al., 2012, 2018). Intensity can be conveyed through all the modes by which emotions are expressed: facial expressions (e.g., broad grins: Barasch et al., 2016); gestures and body language (e.g., banging on a table: Cheshin et al., 2012); text-based communication (e.g., using repated paralingual cues, and/or capital letters: Cheshin et al., 2018); and the voice (e.g., through differences in pitch, stress, or intonation: Banse and Scherer, 1996; Baum and Nowicki, 1998). Variations in intensity may be captured not only by differences within any particular mode (e.g., a glare versus a frown), but often (though not always) by differences in the modes employed (e.g., screaming or banging on a table versus a glare; Cheshin et al., 2012).

Differences in the intensity of emotional expressions may or may not represent the actual intensity of the experienced emotion. Personal goals or situational demands—including organizational display rules—may lead people to exaggerate or suppress their felt emotion, so that their emotional display is not necessarily aligned with their true feelings (Hochschild, 1983; Morris and Feldman, 1996; Grandey, 2000). Here, again, as discussed above, I am concerned with the inappropriateness of the display, not the alignment between the display and the felt emotion. In particular, there are situations where intense emotional displays are non-normative and inconsistent with display rules. In general, high-intensity emotional displays are considered more appropriate in settings where the trigger giving rise to the emotion is apparent and meaningful not only to the individual displaying the emotion, but also to those observing (or targeted by) the display—for example, in settings involving high-stakes conflicts or strong communal relationships (Clark and Taraban, 1991; Frijda et al., 1992; Rose et al., 2006; Lindebaum et al., 2016). In contrast, low-intensity emotional displays are typically considered appropriate in interactions with people one does not know closely, including exchange relationships and most service settings (Cheshin et al., 2018). On the other hand, there are also occasions when the intensity of an emotional display may be *too* low, for instance, someone receiving a highly valued reward, such as winning a gold medal in the Olympics, and displaying only a tiny smile. Shields (2005) describes appropriateness of intensity in emotional displays as based on *emotional borderlines* that define when emotional displays are either excessive or insufficient.

In this vein, the intensity of anger displays has been addressed by Geddes and Callister’s (2007) Dual Threshold Model of Anger, discussed above. Extremely high-intensity anger displays, such as those that involve physical actions (e.g., slamming a door or pounding on a desk), are likely to cross the impropriety threshold in most contexts (Gibson et al., 2009). Such high-intensity expressions of anger yield negative rather than positive outcomes, because they shift the focus from the reason for the anger to the person displaying it. Evidence for the Dual Threshold Model of Anger comes from a large body of literature, of which only a sample is presented here. Cheshin et al. (2012) found that patients and their escorts who displayed “loud” (i.e., high-intensity)—and thus inappropriate—anger were more likely than those who displayed “silent” (i.e., low-intensity) anger to be removed by security staff from a hospital emergency room. Gibson et al. (2009) evaluated anger episodes in six different organizations, and looked at outcomes for the displayer of anger, for the relationship between the displayer and the target, and for the organization. In all cases, the less intense (and therefore more appropriate) the display of anger was assessed to be, the more positive were the consequences across all three outcomes studied. Adam and Brett (2018) found a curvilinear relationship between anger intensity and negotiation outcomes, where concessions from the opposing side rose when moderate anger was expressed, but then fell again when the anger grew in intensity (and was in consequence perceived as less appropriate). Glikson et al. (2019) found that customers’ angry complaints yielded different results based on the intensity with which the anger was displayed. High-intensity anger was seen as both less appropriate and as more threatening than anger of lower intensity. Interestingly, Glikson et al. also found that while high-intensity anger was always deemed less appropriate than low-intensity anger, the outcomes of the anger display depended partly on culture, a finding to which I will return later in this manuscript. Finally, recent work by Staw et al. (2019) showed that the intensity of coaches’ emotional affective displays at half time had a curvilinear impact on team performance in the second half of the game. Performance suffered if the coach’s intensity was either too low or too high, but improved when the coaches’ emotional display was perceived as being at the appropriate intensity level.

Even when it comes to happiness, too much of a good thing can be bad. For example, Barasch et al. (2016) showed that people assess very happy individuals to be more naïve than those who display happiness more moderately, and as more likely to be targeted for exploitation by others. Once again, it is not the intensity *per se* that matters, but the inappropriateness of the display. As Barasch et al. (2016) noted, “the perceived appropriateness of the emotion is likely to matter. For example, if a person just won the lottery or received a substantial promotion, extreme happiness may be especially appropriate and not displaying extreme happiness may be met with negative reactions” (p. 201).

Cheshin et al. (2018) support these findings on happiness intensity while adding sadness to the mix. In a service setting, they examined how displays of happiness and sadness that varied in intensity affected evaluations of service providers and actual use of the product they promoted. They found that differences in happiness and sadness intensity were recognized, whether displayed via the face and body, by intonation, or even merely by text. For both emotions, high-intensity displays were deemed less appropriate than low-intensity displays, and appropriateness mediated the relationship between customers’ assessments of the display intensity and their evaluations of the service and product.

Also with regard to sadness, there is evidence that crying at work—a relatively intense expression of sadness—can have negative consequences for the displayer. For example, in a hospital setting, medical students who cried were ridiculed or berated for their behavior, which was deemed unprofessional (i.e., inappropriate) (Wagner et al., 1997). It has also been found that while crying elicits social support from others, this is often accompanied by negative evaluations of the crying individual (Hendriks et al., 2008; Pauw et al., 2019). Elsbach and Bechky (2017), in the study mentioned earlier, found that episodes in which professional women cry at work are assessed differently based on their “conformance to cognitive scripts that dictate the context and behaviors allowed and prohibited”—i.e., internalized display rules (Elsbach and Bechky, 2017, p. 150). Elsbach and Bechky note that crying was seen as more inappropriate to the degree that it was more intense (e.g., “bawling,” “too emotional,” or “overkill”). Such inappropriate episodes led observers to apply dispositional rather than situational attributions to the crying. For instance, excessive criers were seen as overly emotional, unprofessional, or manipulative, as opposed to reacting normatively in response to a difficult situation.

Much of the work on crying has dealt with gender issues, with crying by males seen as more inappropriate than crying by females, especially in the eyes of other males (Cretser et al., 1982). Studies in sports contexts have found that male players who display low-intensity, moderate crying (e.g., “tearing up”) are perceived as having higher self-esteem compared with players who cry more intensely (Wong et al., 2011; MacArthur and Shields, 2014). Warner and Shields (2009b) examined how the intensity of tears (as opposed to merely their presence or absence) affected evaluations of men and women. They found that men who expressed sadness via low-intensity crying (“a moist eye”) were evaluated more positively than women in similar scenarios, with the tears taken as indicating that the person is sensitive, but has control over their feelings (Warner and Shields, 2009b). However, Vingerhoets and Bylsma (2016), in a review of the relevant literature, argue that the appropriateness of crying, given the context, has a greater impact on the response than the gender of the crying indivdual.

Overall, it is clear that intensity is a key variable in determining the inappropriateness of an emotional display. However, as noted by Barasch et al. (2016); Adam and Brett (2018), and Glikson et al. (2019), more work is needed on the interpersonal effects of emotion intensity (perhaps with the exception of anger). In particular, most work to date deals with cases where high-intensity displays are inappropriate and lead to negative outcomes, leaving open the question of whether and when high-intensity emotional displays are deemed appropriate and are beneficial. Based on the above, I propose the following:

Proposition 4: High-intensity displays of emotion are more likely to be perceived as inappropriate than low-intensity displays.

### The Display Medium

As our world becomes more and more digital, interactions between individuals are increasingly mediated by technology. For example, employees in a range of fields work in virtual teams that are not bound to a specific location, and communicate via digital devices (e.g., Gilson et al., 2015). This section discusses how emotions are displayed in computer-mediated communication as opposed to face-to-face communication and how perceived inappropriateness plays a role. Given the relative newness of this medium, most research in this area still deals with the more basic question of how emotions are displayed in digital communications rather than specific aspects of these displays, such as their inappropriateness.

Despite the relative scarcity of non-verbal cues in computer-mediated communication, evidence for the presence of emotions and emotion dynamics in this medium is robust (e.g., Derks et al., 2008; Cheshin et al., 2011; Baralou and Mcinnes, 2013). Visual cues, such as emoticons and emojis, have evolved as a means to overcome the lack of non-verbal cues in digital communications (e.g., Dresner and Herring, 2010; Stark and Crawford, 2015; Miller et al., 2016; Hu et al., 2017). Yet, people’s ability to recognize emotions and interpret emotional displays in computer-mediated communications has not kept pace with the burgeoning use of communications technology, leaving the emotional content of many messages murky and misinterpretations a constant hazard (Derks et al., 2007; Byron, 2008; Laubert and Parlamis, 2019). Even the length of an email and response times have been taken as emotional cues, and even these have been found to lead to differing conclusions at times (Byron and Baldridge, 2007). Byron (2008) suggests that the creation of display norms can help users interpret emotional content in computer-mediated communication. Indeed, there is evidence that teams using computer-mediated communication develop their own norms of interaction (Postmes et al., 2000; Cheshin et al., 2013). Moreover, Cheshin et al. (2013) showed that while norms may be created based on the specific medium being used (e.g., text messages vs. face-to-face communication), these norms stick even when communication channels change. These findings point to the importance and stability of both communication norms and emotional norms in virtual communications, and hint that violations of these norms will be noticed, and as such should lead to negative consequences.

There are also organizational norms and expectations regarding what one should and should not communicate via phone or email, as opposed to face-to-face. For example, employees should never be fired via email or by phone, only face-to-face. Likewise, emotional expressions that seem appropriate in one mode could be deemed inappropriate in another. Byron (2008), in her work on emotions in email, describes two effects that could impact the interpretation of emotions as appropriate or not: the *neutrality effect*, whereby positive messages seem more “emotionally neutral than senders intend” (p. 312), and the *negativity effect*, whereby such messages are seen as more negative than intended. Both effects stem from the fact that non-verbal cues are limited. Thus, a critique delivered via email may seem harsher than criticism delivered face-to-face. However, while displays of anger or sadness may be perceived as greater in intensity if delivered via email as opposed to face-to-face, displays of happiness may be perceived as lower in intensity. These biases should impact inappropriateness assessments of emotional displays.

In recent work investigating the impact of violating emotional display norms in computer-medicated communication, Glikson et al. (2018) tested how the use of smileys impacts first impressions. They found that unlike a face-to-face smile, which leads to impressions of warmth, use of smileys led participants to perceive new colleagues as less competent; as a result, participants tended to share less information with smiley-users. The driver for these adverse responses was the assessment of smileys as inappropriate in a formal business setting. When the smiley was used in relation to an informal social gathering, the smiley did seem appropriate and the negative outcomes were eliminated. In another recent study, Riordan and Glikson (2019) verified the importance of communication norms in assessing the appropriateness of emojis. In a set of studies, they showed that managers who used emojis in organizations that had formal communication styles were seen as less effective. Li et al. (2019) found similar results in a customer service setting, where the appropriateness of using emojis—defined by the communal or exchange relationship—determined customers’ satisfaction with the service. These studies attest to the impact of inappropriateness of emotional displays in computer-mediated communication—in this case via emojis—on outcomes for the displayer and the organization. Therefore, the following is proposed:

Proposition 5: Violating norms of emotional displays in computer-mediated communication will lead to negative outcomes for the displayer and the organization.

As we have just seen, digital communication adds another layer of complexity to the question of when and where emotional displays are perceived as appropriate. First, the norms of face-to-face communication are not always transferable to digital communication. Further, the nature of a business’s communication style may affect the appropriateness of emotional expressions in electronic communications. Finally, electronic communication allows for easy interaction between people from different national as well as organizational cultures. Glikson and Erez (2013) showed that different cultures have different norms that dictate the (in)appropriateness of emotional displays—e.g., norms for the display of positive and negative emotions—even in computer-mediated communication. It is to differences in cultural norms regarding emotional display rules, and other aspects of the context, that I turn next.

## Characteristics of the Context

Characteristics of the context is a broad category. Its scope ranges from the very narrow (e.g., the topic of the interaction) through the surrounding context (e.g., formal vs. informal) or culture (e.g., a hierarchical vs. flat organizational culture), to the broad (e.g., industry or sector) and very broad (e.g., national culture). Any given context comes with expectations and norms about how one should behave, which of course include emotional display norms. The following brief review highlights contextual differences in the perceived inappropriateness of emotional displays in the workplace, and the consequences of norm violations for the emotional displayer and for the organization.

At its most narrow, the context comprises the topic or purpose of the interaction. For example, apologies are thought to be accompanied by emotional displays of remorse, regret, shame, and sadness. Displays of other emotions are deemed inappropriate, with potentially deleterious effects for the displayer and the organization. ten Brinke and Adams (2015) investigated the organization-level effects of emotion displays during public apologies following revelations of corporate wrongdoing. They found that apologies accompanied by displays of inappropriate emotions, such as happiness, were assessed as less sincere and yielded worse outcomes in terms of investor confidence and stock market returns. Moreover, these effects lasted as long as 90 days after the incident, indicating that the consequences of emotion displays can have a relatively long duration.^5^

At the next level up, permitted or expected emotional expressions tend to vary between formal and informal contexts (e.g., a business environment vs. a social gathering). Broadly speaking, more rules regarding emotional displays operate within work/business settings than outside them (Moran et al., 2013). That is, in work contexts, the space within which emotions can be expressed (i.e., the space between the emotional expression and the impropriety thresholds) is likely to be narrower than in non-work contexts. Organizational cultures can be more or less formal and hierarchical, meaning that standard display rules for formal contexts may be enforced or encouraged to a greater or lesser extent by particular organizations. An organization with a very flat culture may allow greater expressions of emotion than one with a very hierarchical culture (Domagalski, 1999; Matsumoto et al., 2008).

Some emotion display norms vary between different industries or sectors. For example, anger displayed by a service provider will almost always be deemed inappropriate and lead to adverse outcomes for the organization and the individual displayer (e.g., customer dissatisfaction or complaints, and in the worst cases, the offender losing his/her job; Rafaeli and Sutton, 1987; Grandey et al., 2010; Gabriel et al., 2016). However, in non–customer-facing settings, anger can be a useful tool, as seen in findings that construction project managers employ anger to help ensure the progress of the project (Lindebaum and Fielden, 2011) and that displays of anger by military leaders can be considered appropriate and motivating (Lindebaum et al., 2016). Similarly, Gibson et al. (2009) found different organizational norms regarding anger between sectors. One sector that is governed by powerful (if implicit) rules relating to displays of emotions is the legal system, where emotion displays have been found to impact legal decisions. Rose et al. (2006) found that complainants who do not display emotions deemed appropriate to the “victim role” receive less sympathy, and offenders in those cases receive lesser punishment; while Heath (2009) provides examples of cases where emotional displays that were deemed unfitting and non-normative led to arrests and even convictions of potentially innocent defendants in the United States.

With respect to national culture, a large body of literature has examined the effect of different cultural values on emotional display norms. Some organizational or sector norms transcend national boundaries. For instance, Grandey et al. (2010) found that emotional display norms with respect to customer service are fairly consistent across the globe. However, in many cases, such norms diverge based on national culture. To cite just one example, in Singapore, it is considered less acceptable to display anger and sadness than in the United States (Moran et al., 2013). Glikson and Erez (2013) observed that different emotional display norms emerged in virtual teams when the groups were culturally homogeneous, implying that such norms differ between the five countries they examined. Much of the literature on culture and emotion relies on the classic distinction between individualistic and collectivistic cultures. In general, this work has found that anger displays, in particular, are deemed inappropriate in collectivist cultures, where expressing anger poses a threat to group harmony, whereas in individualistic cultures, anger displays may be appropriate in different circumstances (e.g., Kitayama et al., 2006). Another cultural value found to affect assessments of inappropriateness is power distance—i.e., one’s acceptance for power inequalities and social hierarchies (Hofstede, 2001). Glikson et al. (2019) mentioned above, found that displays of high-intensity anger by customers were perceived as less appropriate by service providers (the target of the anger) who scored low (vs. high) in power distance. This led to lower amounts of compensation for the angry customer.

Negotiation settings offer a profitable vantage point from which to examine the effects of culture and context on emotional expressions. A large body of work has examined the effects of displaying emotions—especially anger—in negotiations, both within cultures and cross-culturally. To cite just a few examples: Kopelman and Rosette (2008) found that Israelis were more likely to accept an offer from counterparts when the offer was accompanied by negative emotions, while East Asians were less likely to do so. This has been attributed to differences in cultural norms relating to humility and deference between Israelis and East Asians. Adam et al. (2010) attest to differences in responses to anger in negotiations between Americans of European ancestry and people of East Asian backgrounds. The European Americans conceded more to an angry opponent while the Asians conceded less, with the responses explained by assessments of the anger as (in)appropriate. Other studies have examined the effects of anger in negotiations on the basis not of culture, but of other contextual features. For instance, Adam and Brett (2015) found that anger leads to positive outcomes for the displayer in competitive negotiations and negative outcomes in cooperative negotiations, with hints that the appropriateness of the display is the mechanism involved. Similarly, van Kleef and Côté (2007) found that manipulating norms regarding anger and indicating when a display is appropriate and when it is not, determined how people reacted to an angry counterpart in a negotiation. Outcomes for the negotiators were better when the anger was perceived as appropriate.

Sadness has received attention in negotiation research. In one study, displays of sadness led to greater concessions from the other side, presumably because the latter felt concern for the person displaying sadness (Sinaceur et al., 2015). In one of their experiments, Sinaceur et al. (2015) examined the interaction between the emotion displayed (anger or sadness) and the appropriateness of the emotion. In that experiment, the negotiating partner who was exposed to the emotional display was informed either that in negotiations it is inappropriate to blame the other side for disagreements or that blaming others was a normal and natural part of negotiations. Sinaceur et al. (2015) found that in the conditions where blaming was deemed inappropriate, participants who displayed sadness, which is not indicative of blame, obtained better outcomes in the negotiation than those who displayed anger, which is indicative of blame. However, when blaming the other side was deemed to be the norm, sadness displays did not lead to better outcomes for the displayer. Thus, only when sadness was seen as more appropriate than anger did it lead to positive outcomes for the displayer.

Rees and Kopelman (2019) offer a similar argument, contending that appropriateness rather than rationality is what drives success in cross-cultural negotiations. They argue that actions that seem rational and “make sense” but are inappropriate result in poor outcomes, while those that seem irrational but appropriate result in good outcomes. Thus, in the intersection between culture and emotion, the logic of appropriateness (based on norms) trumps the logic of rationality (based on reason) (see also Kopelman, 2009; Kopelman et al., 2016).

Indeed, in light of the full range of the literature covered in this section, it is likely that this conclusion holds for the full range of settings, contexts, and characteristics discussed here. For example, with respect to anger, it might be considered reasonable and appropriate to show anger when one wants to be seen as tough and resolute, or when the situation is dear to one’s heart, but this could backfire in a cooperative setting, or where the cultural norm calls for suppression of emotions. With respect to happiness, it might seem reasonable to incorporate a smiley as a substitute for a (real) smile when sending an email to a new work colleague, yet this could be perceived as inappropriate and unprofessional, and possibly even a sign of lower competence. Finally, with respect to sadness, it might be reasonable for a manager to display sadness after failing to meet a goal, but an excessive show of unhappiness—especially by a male manager—would likely seem inappropriate to others, and lead to perceptions of the manager as weak or less competent.

Based on the above, I propose the following:

Proposition 6: Assessments of emotional displays as inappropriate will differ based on the context and culture.

Table 1 offers examples of how features of emotional displays—characteristics of the displayer, the display, or the context—can lead to negative outcomes for the person displaying the emotion, or for the organization. The examples incorporate anger, sadness, and happiness.

**TABLE 1:** Examples of negative outcomes following inappropriate displays of emotion.

	**Outcomes for Displayer**	**Outcomes for Organization**
**Characteristics of Displayer**		
Status	Subordinates are more likely to be punished or sanctioned for anger displays than supervisors (Fitness, 2000)	Subordinates who expressed anger report less positive outcomes (compared to supervisors) related to situational problem improving and relational problems (Callister et al., 2017)
Gender	Male supervisors are assessed as less effective when displaying sadness compared to neutrality (Lewis, 2000)	Expressions of anger by females led to more negative organizational outcomes than males (Gibson et al., 2009)
**Characteristics of Display**		
Intensity	High-intensity happiness and sadness shown by service providers led the service provider to be assessed as less trustworthy (Cheshin et al., 2018)	A product was assessed worse and was less likely to be used when the intensity of happiness and sadness of a service provider was high rather than low (Cheshin et al., 2018)
Mode of Communication	Using smileys in first-impression formal email communications led to lower assessments of competence (Glikson et al., 2018)	A company’s service was deemed worse by customers when it included a smiley in exchange relationships (Li et al., 2019)
**Characteristics of Context**		
Topic	Apologies for corporate wrongdoing by CEOs were assessed as less sincere when accompanied by displays of inappropriate emotions, such as happiness (ten Brinke and Adams, 2015)	Apologies for corporate wrongdoing were assessed as less sincere when accompanied by displays of inappropriate emotions, such as happiness, and yielded worse outcomes in terms of investor confidence and stock market returns (ten Brinke and Adams, 2015)
Culture	High-intensity anger displays by customers were perceived as less appropriate by service providers based on cultural values (high power distance), leading to lower compensation following complaints (Glikson et al., 2019)	Anger expressions in collectivist cultures are assessed as inappropriate and pose a threat to group harmony compared to anger expressions in individualistic cultures (Kitayama et al., 2006)

## Boundary Conditions for the Current Model

### Authenticity

As mentioned above, emotions can be displayed even if they are not genuinely felt by the individual. At times, this is done intentionally to adhere to display norms and to try to display an emotion that is appropriate. However, I see this as a separate issue that, no doubt, has an impact on outcomes of emotional displays (e.g., Tng and Au, 2014; Gabriel et al., 2015; Hideg and van Kleef, 2017). This is a different, and important, aspect of emotional display that has been addressed by others. There is no doubt that the emotion display will lead to better outcomes when it is both authentic and appropriate.

### Containment of Inappropriateness

Geddes and Stickney (2011) found that some organizations react to deviant displays of anger by offering support rather than sanctions. That is, instead of punishing “emotional outlaws,” these organizations encourage managers or coworkers to approach the angry employee in a mode of supportive concern. Geddes and Stickney found that offering such support leads to positive change and improved outcomes for the employee and the organization. Thus, in such cases, inappropriate behavior that could be expected to have negative outcomes is turned around so that the outcomes are positive. Such transformations require both vigilance and a proactive approach by the organization.

Similarly, organizations may be able to reverse the negative effects that might follow inappropriate displays of other emotions, such as sadness or happiness, though the approach may be different in each case. Anger is known to arise when a goal is obstructed or an injustice is observed (Smith and Ellsworth, 1985; Frijda, 1986). Supportive behavior may resolve the problem in part by addressing the problem that gave rise to the anger. Sadness is likewise a negatively valenced emotion that tends to arise when there is something wrong, and addressing the cause of the sadness or supporting the displayer may help reverse or prevent negative outcomes from the display. When a positively valenced emotion such as happiness is displayed in an inappropriate manner, the implication may be that the person displaying the emotion did not understand the norm. Thus, a successful response to such displays may entail providing clearer guidelines as to display rules. However, in all these cases, the effectiveness of a supportive response may depend on another dimension that I have not yet discussed: whether the inappropriate display crosses the bounds of civility.

### Two Distinguishable Forms of Perceived Inappropriateness of Emotional Display

According to the Dual Threshold Model of Anger (Geddes and Callister, 2007), when the impropriety line is crossed—anger is deemed as inappropriate and deviant. Deviant anger is “damaging, and/or unacceptable given the circumstance” (p. 732). I interpret this to mean that anger that has been deemed as deviant will always lead to negative outcomes for the displayer. It is clear, however, that the impropriety threshold can shift and move between cultures and contexts, yet once an anger display is seen as deviant, it would not be accepted and/or would be damaging.

Recently, Lindebaum et al. (2016) point to a seemingly paradoxical sentiment toward anger in the military. On the one hand, display rules in the military aim to reduce anger, as part of the military’s effort to curtail its traditional bullying culture. On the other hand, there are tasks and situations in the military that require anger to be displayed. In that study, expressions of anger, even extreme expressions that included shouting and cursing, were seen as positive by (some of) the targets of the anger, who made sense of the anger as necessary due to the circumstances and assisting in the task (e.g., by signaling urgency). Thus, despite being rude and uncivil, the anger displayed was deemed appropriate. This is an example where an extreme display of anger is not deviant, as it does not lead to damage, nor is it seen as unacceptable; on the contrary, it is deemed appropriate to the situation—fitting the context.

The point of interest here is that in Lindebaum et al. (2016) study, extreme expressions of anger were seen as acceptable and appropriate even though such expressions could be described as impolite, uncivil, and rude. Thus, impropriety is to be distinguished from incivility. Incivility (i.e., behaving in an uncivil manner) is defined as “acting rudely or discourteously, without regard for others, in violation of norms for respect in social interactions,” or, in the workplace, “in violation of workplace norms for respect” (Andersson and Pearson, 1999, p. 455; see also Pearson and Porath, 2009). A display of emotion can be uncivil or rude and still be deemed appropriate. Likewise, it can be civil and courteous but still deemed inappropriate based on characteristics of the displayer, the display, or the context, as discussed above. Adopting this notion, I would like to propose that inappropriateness can take on two forms—*civil* and *uncivil*.

Inappropriateness should be distinguished and divided into two different forms. One is uncivil-inappropriateness, meaning that this inappropriateness display is rude, and therefore negative and harmful. The other is civil-inappropriateness, meaning the display is odd and non-normative, yet considered polite. An uncivil-inappropriate emotional display could be analogous to an anger display that crosses the impropriety threshold of the Dual Threshold Model of Anger (Geddes and Callister, 2007). Thus, I propose that it is when one crosses the line and is extremely inappropriate and rude that the outcome will be negative, whereas a perceived *civil*-inappropriate emotional display, which is not deemed rude, and although inappropriate—does not cross the “civility” line—could lead to a more rational and level-headed response.

The EASI model suggests that responses to the emotional displays of others will be based more strongly on affective reactions (as opposed to inferential processes) to the degree to which the emotion encountered is perceived as inappropriate (van Kleef et al., 2012; van Kleef, 2014). It is argued the negative affective reactions that follow inappropriate emotional displays overwhelm any concurrent inferential processes (van Kleef, 2014). The distinction between inappropriateness and incivility raises the question of whether the prioritization of the affective route may be a function of incivility rather than inappropriateness, at least in some cases.

For example, Cheshin et al. (2018) showed that customers in a service setting interpreted high-intensity displays of happiness or sadness by a service provider through the inferential route. It may be that in this case, it is because the inappropriate display was not perceived as uncivil that the (negative) affective route did not take precedence. Another example can be found in Glikson et al. (2018). When a smiley was used in a first impression email, it was deemed inappropriate, which led the targets to respond to it less favorably. This response was due to assessing this writer of this email as less competent (inferential route) and probably not due to an affective reaction to the inappropriate smiley.

An example of where behavior can be uncivil yet appropriate can be found in competitive behavior of trash-talking. A recent article by Yip et al. (2018) demonstrated how trash-talk, or “boastful comments about the self or insulting comments about an opponent that are delivered by a competitor typically before or during a competition” (p. 126), impacts individuals and organizations. This uncivil act was found to be common in competitive settings and has been found to motivate the targets to put forth more effort on competitive tasks. However, in cooperative settings, this uncivil act of trash-talk harmed performance. The authors state that: “Some forms of trash-talking are likely to be more appropriate than other forms, and appropriateness may moderate the effects of trash-talking” (p. 140). This is an example of how uncivil acts could be more appropriate in specific setting and not in others.

Thus, inappropriateness takes on two different forms. When it is deemed uncivil, it is a different type of inappropriateness and is similar to other rude and uncivil behaviors in the workplace (see Porath and Erez, 2007; Schilpzand et al., 2016) and, as such, evokes negative affective reactions. More simply, inappropriate behavior that is also uncivil and rude may be treated much like any other kind of uncivil, rude, or discourteous behavior. Yet, expressions of emotion that are non-normative but remain civil, courteous, and respectful to others may be perceived simply as examples of benign but odd behavior that require further inquiry (Stern et al., 1984), leading observers to react via the inferential route.

In short, it is suggested here that it is not inappropriateness *per se* that leads to (negative) affective reactions, but rather the intersection between (in)appropriateness and (in)civility. An emotional display that is deemed inappropriate but civil may have a negative outcome, based on the inferences drawn from the display. However, these claims need to be empirically examined. As a suggestion to launch future studies, Table 2 provides examples of the interaction between whether an emotional display is appropriate or inappropriate and whether it is civil or uncivil, along with (1) the likely route by which displayed emotions influence observers (inferential or affective) and (2) the likely outcome (positive or negative).

**TABLE 2:** Interaction between (In)appropriateness and (In)civility in emotional displays.

	**Inappropriate Emotional Display**	**Appropriate Emotional Display**
UNCIVIL	*Example*: Patient/escort shouting and cursing a nurse in the ER. *Process*: Affective (negative). *Outcome*: Security is called to remove the patient/escort from the ER. Negative outcome for the displayer.	*Example*: Drill sergeant berates cadets for failing to meet standards. *Process*: Inferential. *Outcome*: The cadets understand they have violated expectations and correct their actions. Positive outcome for the displayer and the organization.
CIVIL	*Example*: New colleague closes an email with a smiley. *Process*: Inferential. *Outcome*: The new colleague is assessed as less competent, and less information is shared with him/her. Negative outcome for the displayer, and possibly the organization.	*Example*: Service provider smiles in accordance with display rules. *Process*: Inferential and/or affective. *Outcome*: The service provider and firm are evaluated positively.

Based on the above, I propose:

Proposition 7: Reactions to an emotional display will depend both on whether the display is appropriate or inappropriate and whether it is civil or uncivil. When the inappropriateness is also uncivil, the affective reaction will be negative and will dominate the response and outcome to the displayer; yet, when the inappropriateness is civil, it will lead to (negative) inferences that will dominate the response and outcome to the displayer.

## Discussion and Conclusion

Contrary to the everyday belief that emotions are evaluated as positive or negative based on the valence of the emotion, in many cases, it is actually the perceived inappropriateness of the presentation that determines how it is evaluated by observers, or by the target of the emotion display. Emotional displays that are perceived as inappropriate may give rise to negative outcomes for the displayer, organization, or both. Indeed, the review presented in this paper shows that the (in)appropriateness of emotional displays has vast implications that reach far beyond the impact of the discrete emotion displayed. What Rees and Kopelman (2019) argue regarding multicultural negotiations is true not only in negotiations but also in multicultural interactions. Rather, the landscape of display norms and rules guides perceptions of (in)appropriateness for emotional displays in any setting. This landscape includes the characteristics of the displayer, the display, and the context in which the display occurs.

The present paper builds on the Dual Threshold Model of Anger (Geddes and Callister, 2007) and applies the impropriety threshold to all emotion displays. Furthermore, the paper continues the line of work of Lindebaum and Jordan (2014) and van Kleef (2014), suggesting that discussions of emotional processes have been oversimplified, with most attention paid to symmetrical effects. In addition, this review shows that inappropriateness of emotional displays plays a role not only via the affective route (i.e., in the emotions elicited by the display), but also via an inferential route—i.e., through the cognitive inferences people draw from emotion displays. Lastly, the present manuscript suggests that (in)appropriateness intersects with (in)civility to engage either the affective or inferential route. The affective path dominates when a display crosses both the impropriety threshold (i.e., violates display rules) and violates the norms of etiquette. The inferential path dominates when the display crosses the impropriety threshold but remains within the bounds of etiquette and civility.

At a practical level, this review suggests that organizations could benefit from addressing the (in)appropriateness of emotion displays. For example, human resource management should consider how to train and guide employees to follow display rules on the basis of what features of a display are (or are not) appropriate (Gabriel et al., 2016). Geddes et al. (2019), in their recent work on anger, have called for organizations to offer “appropriate space” to express anger, which could take advantage of the positive aspects of anger expression. The idea of a place where anger is welcomed as appropriate symbolizes the essence of the present paper. When emotional expressions have a place and are seen as appropriate and fitting, the outcomes—for the person experiencing the emotion and for the organization—should be positive. Along these lines, Grandey et al. (2015) have called for organizations to eradicate display rules completely, on the grounds that their costs outweigh their benefits. The idea is that authentic displays and a positive climate will be beneficial for all. However, this view does not take into account others’ expectations. In the realm of customer service, for example, display rules reflect the fact that consumers expect a certain deference and professionalism from service providers. Authentic displays of emotion by service providers could lead to unsatisfied customers who take their business elsewhere.

The present work is subject to limitations, some of which offer potentially fertile ground for further research. First, despite the evidence reviewed here, more work is needed to establish the claims raised in this paper. In this respect, the Perception of Emotion Appropriateness Rating Scale (PEARS), developed by Warner and Shields (2009a), is a validated measure of emotional appropriateness that could be useful in future research to validate ideas presented in this manuscript. Second, in the study of emotions, anger has dominated the literature. This makes sense, as anger potentially has the most detrimental effects on individual and organizational outcomes (including the potential to turn into aggression and violence). But this focus on anger means that less is understood about the dynamics of other emotion displays in the workplace. Future research should concentrate and focus more closely on other discrete emotions, including but not limited to happiness and sadness. Moreover, incivility will, by nature, most often apply to anger. But other discrete emotions can also be viewed through this lens. Research should examine whether and when displays of emotions other than anger can be perceived as uncivil and rude (e.g., loud and incessant laughter or crying in a public place), or whether there are discrete emotions that are “immune” from the risk of incivility. Third, instances where display intensity is high yet perceived as appropriate are somewhat lacking. Likewise, the distinction between intensity and incivility in emotional displays could benefit from clarification. At what point does an intense display of (for example) sadness in the workplace cross the boundaries of etiquette (as well as workplace display norms) and become not only inappropriate, but also rude? Fourth, it should be noted that, at times, observers or targets of an emotional display may fail to accurately identify the emotion (or combination of emotions) being displayed (Fang et al., 2018). This is in part because some emotional expressions overlap with others. A good example is the smile. While a smile is considered a basic display of happiness, people are also known to smile when they are embarrassed, fearful, contemptuous, angry, dominant, submissive, listening, and more (Hess et al., 2002; Beukeboom, 2009; Perron et al., 2016). As such, the way an observer interprets a display of emotion may not be entirely aligned with the feelings of the displayer. It has also been shown that people are able to recognize more than one emotion in others’ displays (Fang et al., 2018). Thus, it may be simplistic to assume that during interpersonal exchanges, people encounter and respond to one discrete emotion. Yet, most work to date does tend to focus on the evaluation of discrete and specific emotions, distinguishing between features of each emotion separately. Future work should take this element into account.

Another point to stress is that norms and customs change over time. For example, gender differences in the workplace have softened and blurred considerably over recent decades. This trend will probably continue, further altering norms about behavior perceived as (in)appropriate, for women and men. Likewise, continuing advancements in technology will doubtless affect what kinds of emotional displays are deemed appropriate in computer-mediated communication. The relatively new use of emojis could make behaviors that appear inappropriate in 2020 (e.g., the use of smileys in business contexts) not only acceptable but desirable in the future. In this vein, testing and conceptualizing the different contexts and settings in which emotion displays operate can offer challenging and exciting avenues for research. For example, would displays of emotions by bots, or other forms of artificial intelligence, also be impacted by perceptions of inappropriateness? Imagine getting emotional feedback from your cellphone (e.g., happy squeals or a self-satisfied sigh). Would the same judgments regarding inappropriateness apply as in human conversations?

Overall, this manuscript shows that evaluating interpersonal aspects of emotions in terms of valence alone, as either “good” or “bad,” is insufficient. Only after taking account of inappropriateness in emotional expressions can one evaluate the true valence of the emotion, in terms not only of how we feel but how the emotion affects our surroundings and others’ responses to us.

## Author Contributions

The author confirms being the sole contributor of this work and has approved it for publication.

## Conflict of Interest

The authors declare that the research was conducted in the absence of any commercial or financial relationships that could be construed as a potential conflict of interest.

## Footnotes

1 It is important to note that, in some situations, failing to display an emotion could be deemed an inappropriate reaction (Warner and Shields, 2009a). Such situations are beyond the scope of the present study, which focuses on actual interpersonal displays of happiness, sadness, and anger.

2 According to Thoits (2004), however, such emotional deviants may become agents for social change. The present paper does not concern itself with such possible long-term outcomes.

3 This is by no means a claim that the communication medium or the expression form has no impact on inappropriateness. On the contrary, the communication medium is one of the characteristics of the display that impact inappropriateness as well as the expression form. The point is that an emotional display attempts to convey the same meaning to others regardless of the manner in which it is displayed, and if deemed appropriate, the display represents the same emotion and, as such, will be assessed in a similar manner and will lead to a similar outcome. For example, displaying anger via tone of voice or by facial displays would lead to similar outcomes when they are displayed appropriately, however, when the anger displayed is deemed inappropriate, the response would be different. The same goes for a smiley; if it is perceived as appropriate, it should have a functionally equivalent outcome to a display of happiness via body, tone of voice, face, or text.

4 See, however, Elsbach and Bechky (2017), who found that observers applied both situational and dispositional attributions to professional women who cried at work, depending on whether the behavior fit the context and observers’ expectations.

5 In addition, when the apology was made by a higher-status individual (the CEO), the effect of the apology was stronger than when it was made by a lower-status organizational member.
